# Global methaemoglobinaemia research output (1940–2013): a bibliometric analysis

**DOI:** 10.1186/s40064-015-1431-7

**Published:** 2015-10-19

**Authors:** Sa’ed H. Zyoud, Samah W. Al-Jabi, Waleed M. Sweileh, Suleiman Al-Khalil, Malik Alqub, Rahmat Awang

**Affiliations:** Poison Control and Drug Information Center (PCDIC), College of Medicine and Health Sciences, An-Najah National University, Nablus, 44839 Palestine; Department of Clinical and Community Pharmacy, College of Medicine and Health Sciences, An-Najah National University, Nablus, 44839 Palestine; WHO Collaborating Centre for Drug Information, National Poison Centre, University Sains Malaysia (USM), 11800 Gelugor, Penang Malaysia; Department of Pharmacology and Toxicology, College of Medicine and Health Sciences, An-Najah National University, Nablus, 44839 Palestine; Department of Anatomy, Biochemistry and Genetics, College of Medicine and Health Sciences, An-Najah National University, Nablus, 44839 Palestine

**Keywords:** Bibliometric, Citations, *h*-index, Methaemoglobinaemia, Scopus database

## Abstract

Bibliometric studies, which involve the use of statistical methods, are increasingly being used for research assessment. A bibliometric analysis was conducted to evaluate the publication pattern of methaemoglobinaemia research output at the global level based on the Scopus database. We analysed selected documents with “methemoglobinemia”, or “methaemoglobinaemia” as a part of the title and reported the following parameters: trends of publication output, country of publication, journal pattern, collaborative measures, citations pattern, and institute productivity. A total of 1770 articles were published worldwide. The time trend for the number of articles showed an increase after 2000. The highest number of articles related to methaemoglobinaemia was from the USA (24.8 %), followed distantly by the UK (4.5 %), India (3.7 %), and France (3.7 %). No data related to methaemoglobinaemia were published from 152 countries. The total number of citations at the date of data collection was 10,080, with an average of 5.7 citations per document. The USA and UK had the highest *h*-index of 31 and 14, respectively, and six countries had an *h*-index of 9–14. It is notable that Canada was ranked eighth in the number of publications but fourth in *h*-index and India was ranked third in the number of publications but eighth in *h*-index. Furthermore, Canada produced the most internationally collaborated papers out of the total number of publications for each country (16.1 %), followed by the UK (13.9 %). This bibliometric analysis provides data contributing to a better understanding of the methaemoglobinaemia research field. The number of publications on methaemoglobinaemia increased significantly after 2000. The USA was the most productive country as measured by total publications. The USA and UK achieved the highest *h*-index in the field of methaemoglobinaemia research, signifying a higher quality of research than other countries.

## Background

Methaemoglobinaemia is a disorder that occurs when haemoglobin in the blood is oxidized to form methaemoglobin (MetHb), rendering it unable to transport oxygen. Furthermore, when the MetHb concentration is elevated in red blood cells, it leads to tissue hypoxia (Skold et al. 2011; Cortazzo and Lichtman 2014). Patients are particularly susceptible to worsening methaemoglobinaemia in the presence of oxidizing agents (Mitsides et al. 2014; Sohn et al. 2014; Wieringa et al. 2014). Medications or agents such as anaesthetics (e.g. lidocaine), antibiotics (e.g. sulphanilamide and dapsone), nitrites (e.g. nitroglycerin/nitric oxide), hair dyes, and curing salt are the most likely causative agents for Methaemoglobinaemia (Cortazzo and Lichtman 2014; Mitsides et al. 2014; Wieringa et al. 2014).

There is an individual variation to oxidize Hb, and this may lead to variations in responses to ingestion of the previously mentioned agents (Cortazzo and Lichtman 2014). Healthy people can tolerate low levels of MetHb without difficulty (Skold et al. 2011). Cyanosis and hypoxia are observed often due to reduction in peripheral oxygen saturation measurements (Skold et al. 2011), so an increase in MetHb can lead to dyspnoea, headache, dizziness, seizures, acidosis, arrhythmias, coma, and death (Skold et al. 2011; Cortazzo and Lichtman 2014). Early recognition of methaemoglobinaemia is important because removal or stopping of the causative agent is the first line in treatment (Skold et al. 2011).

Bibliometric studies, which involve the use of statistical methods, are increasingly being used for research assessment (Wallin 2005; Zyoud et al. 2014b, c, d). Methods used in bibliometric studies are mainly quantitative, but can be used to assess the quality of research productivity for a particular country or institution (Wallin 2005; Abramo et al. 2009; Smith 2012). Several recent studies have analysed the scientific research productivity related to various diseases (Sweileh et al. 2014a, b, 2015a, b, c). In point of fact, the assessment of scientific research productivity in the field of haematology has been poorly investigated to date, and only a small number of international bibliometric studies have been published within the field of haematology (Morimoto et al. 2003; Acevedo et al. 2014). To the best of our knowledge, no study until now has been concerned with the assessment of research productivity in the field of methaemoglobinaemia originating worldwide. The objective of the presented study was to analyse the publication pattern of methaemoglobinaemia research output at the global level to provide an accurate overview of scientific output over time and to develop future strategies for research in this area.

## Methods

### Search strategy

The search strategy used in this study was similar to most recent bibliometric studies from our research group (Zyoud et al. 2014a, b, c, d, 2015a, c). Data were obtained from the online version of the SciVerse Scopus. This scientific database embraces the core academic journals related to the subject of interest (Smith 2009, 2010), and it also has a high number of journals in comparison to PubMed and Web of Science (Falagas et al. 2008).

The search conducted in Scopus covered papers published during all the previous years and up to 31 December 2013 whose title included the following keywords: “methemoglobinemia”, and “methaemoglobinaemia”. The resulting search was as follows: TITLE (“methemoglobinemia”) OR TITLE (“methaemoglobinaemia”). We excluded documents that were published as an erratum.

The collected data were analysed to create the following information: year of publication, subject categories, publication counts of the journals, citations received by the publications, collaboration among different countries, assessing research in terms of the most prolific institutions, and impact factors of the publishing journals. Bibliometric indicators were presented in rank order using the standard competition ranking (Zyoud et al. 2014a, b, c, d, 2015a, c). Only the ten top-ranked were considered and interpreted. The quality of research productivity was measured using the *h*-index, impact factor (IF), and the SCImago Journal Rank (SJR) indicator. Impact factors were taken from the Journal Citation Report (JCR) published in 2012. SJR was taken fromthe SCImago website (Scimago 2014). The *h*-index was introduced by Hirsch (Hirsch 2005) as an alternative to the impact factor to characterize the significance of a scientist’s research publications. The *h*-index is the largest number *h* such that *h* papers have at least *h* citations (Hirsch 2005). For example, an *h*-index of eight means that the author has eight papers with at least eight citations each.

### Statistical analysis

All statistical analyses were performed using SPSS software (version15.0; SPSS, Chicago, IL).We used Pearson’s correlation analysis to determine significant changes in publication number over time. All data are expressed as medians and interquartile ranges (IQRs) or numbers with percentages.

## Results

As a result, 1770 publications were obtained. Of all the publications retrieved from the database, original articles accounted for 76.3 %, followed by letters to the editor (10.1 %), review articles (4.0 %), and others (e.g. editorial material, meeting abstract and book review; 9.7 %). Publications in the field of methaemoglobinaemia were seen as early as 1940s followed by a gradual increase that reached the maximum after 2000 (Table 1). Pearson’s correlation test showed a significant, strong positive correlation between time and number of publications (r = 0.88, *P* < 0.001) between 1940 and 2013. Around 33.2 % of all retrieved publications were published during 2000–2013, with a steady publication rate of around 42 documents per year overall (Table 1). The first article related to methaemoglobinaemia in Scopus was published by Carey and Wilson in *The Journal of Pediatrics* in 1940(Carey and Wilson 1940). The language in which the publications were written was predominantly English (n = 1264, 71.4 %), followed by French (n = 106, 6 %), German (n = 83, 4.7 %), and Spanish (n = 56, 3.2 %).

**Table 1:** Total number of articles included in a bibliometric analysis of worldwide publications related to methaemoglobinaemia from 1940 to 2013

Year of publication	Total n = 1770 (%)
1940–1949	29 (1.8)
1950–1959	98 (5.4)
1960–1969	218 (12.3)
1970–1979	246 (13.9)
1980–1989	276 (15.6)
1990–1999	315 (17.8)
2000–2009	389 (22.0)
2010–2013	199 (11.2)

The retrieved documents were published in 60 countries. In Table 2 we see the ten most productive countries in the field “methaemoglobinaemia”. The highest number of articles related to methaemoglobinaemia was from the USA (24.8 %), followed distantly by the UK (4.5 %), India (3.7 %), and France (3.7 %) (Table 2). Among 212 different countries or territories registered in the World Bank online database (World Bank Group 2013), 152 (71.7 %) countries had not published any independent publications related to methaemoglobinaemia.

**Table 2:** The top ten most productive countries with regard to publishing articles related to methaemoglobinaemia

SCR^a^	Countries	Total number of articles for the whole period (%)	*h*-index	Median (Q1–Q3) of citation	Average citation	Collaboration with other countries^b^	Number (%)^c^ of documents with international collaboration^d^
1st	USA	438 (24.8)	31	6 (2–12)	11.2	18	26 (5.9)
2nd	UK	79 (4.5)	14	5 (1–11)	11.5	9	11 (13.9)
3rd	India	66 (3.7)	7	1 (0.0–4)	3.3	7	7 (10.6)
3rd	France	66 (3.7)	9	1 (0.0–3.5)	3.8	2	2 (3.0)
5th	Japan	62 (3.5)	11	1 (0.0–8)	5.8	5	6 (9.7)
6th	Germany	53 (3.0)	10	1 (0.0–8.5)	4.6	3	5 (9.4)
7th	Turkey	47 (2.7)	6	1 (0.0–3.0)	2.2	2	4 (8.5)
8th	Canada	31 (1.8)	10	7 (2–15)	10.4	4	5 (16.1)
9th	Israel	24 (1.4)	9	5 (0.3–10.8)	11.3	1	1 (4.2)
10th	Australia	21 (1.2)	5	3 (0.0–5)	5.1	4	2 (9.5)
10th	Spain	21 (1.2)	5	1 (0.0–4)	4.6	2	2 (9.5)

*SCR* standard competition ranking, *Q1*–*Q3* lower quartile–upper quartile

^a^Equal countries have the same ranking number, and then a gap is left in the ranking numbers

^b^The term “collaboration with other countries” refers to the number of other countries represented among authors were collaborated with authors from a particular country

^c^Percentage of documents with international authors out of the total number of documents for each country

^d^The term “international collaboration” refers to the number of published articles from a particular country and were co-authored by researchers from multiple countries

The total number of citations at the date of data collection (30 January 2014) was 10,080, with an average of 5.7 citations per document and a median (interquartile range) of 1(0.0–6). The *h*-index for all publications was 40 (i.e., 40 documents had been cited at least 40 times at the date of data collection). The USA and UK had the highest *h*-index of 31 and 14, respectively, and six countries had an *h*-index of 9–14. It is notable that Canada was ranked eighth in the number of publications but fourth in *h*-index, and India was ranked third in the number of publications but eighth in *h*-index. Furthermore, Canada produced the most multinational collaborated papers out of the total number of publications for each country (16.1 %) followed by the UK (13.9 %); (Table 2).

Articles were published in 859 journals. Table 3 lists the ten most productive journals with both IF and SJR. Twenty-eight documents (1.48 %) were published in the *Journal of Pediatrics*, whereas 24 (1.36 %) were published in the *New England Journal of Medicine*, 20 (1.13 %) were published in *Blood*, and 20 (1.13 %) were published in *Anesthesiology*.

**Table 3:** Ranking of the top ten journals in which articles related to methaemoglobinaemia were published with their corresponding impact factors

SCR^a^	Journal	Frequency (%)	SJR	IF (2012)^b^	Subject categories^c^
1st	*Journal of Pediatrics*	28 (1.58)	1.2	4.035	Paediatrics
2nd	*New England Journal of Medicine*	24 (1.36)	10.16	51.658	Medicine, general and internal
3rd	*Blood*	20 (1.13)	4.55	9.060	Haematology
3rd	*Anesthesiology*	20 (1.13)	2.05	5.163	Anaesthesiology
5th	*Pediatrics*	15 (0.85)	2.54	5.119	Paediatrics
6th	*Journal of Emergency Medicine*	14 (0.79)	0.47	1.331	Emergency Medicine
7th	*Annals of Emergency Medicine*	13 (0.73)	1.4	4.285	Emergency medicine
7th	*Orvosi Hetilap*	13 (0.73)	0.16	NA	Medicine^d^
9th	*Annals of Pharmacotherapy*	12 (0.68)	0.82	2.567	Pharmacology and pharmacy
9th	*Journal of the American Medical Association*	12 (0.68)	4.84	29.978	Medicine, general and internal
9th	*British Medical Journal*	12 (0.68)	2.327	17.215	Medicine, general and internal
9th	*Toxicology and Applied Pharmacology*	12 (0.68)	1.33	3.975	Pharmacology and pharmacy; toxicology

*SCR* standard competition ranking, *SJR* SCImago Journal Rank, *NA* not available, *IF* impact factor

^a^Equal journals have the same ranking number, and then a gap is left in the ranking numbers

^b^The impact factor was reported according to the Institute for Scientific Information (ISI) journal citation reports (JCR) 2012

^c^Subject categories were reported according to the ISI JCR 2012

^d^Subject categories were reported according to the SCImago Web site

The ten most cited papers of the found bibliography are presented in Table 4 (Curry 1982; Barker et al. 1989; Nilsson et al. 1990; Mansouri and Lurie 1993; Coleman and Coleman 1996; Fan and Steinberg 1996; Wright et al. 1999; Ash-Bernal et al. 2004; Fewtrell 2004; Guay 2009). According to this list, emergency medicine, environmental and toxicological aspects, and drug safety have mainly captured the attention of the researchers throughout the period of publication. The top ten institutions were ranked by the number of articles (Table 5). Among the top ten institutions, six were in the USA. All the top institutions appeared in the top ten ranking countries, except Brazil and China.

**Table 4:** Top ten cited documents related to methaemoglobinaemia in scopus

SCR	Authors and year of publication	Source title	Cited by
1st	Wright et al. (1999)	*Annals of Emergency Medicine*	250
2nd	Coleman and Coleman (1996)	*Drug Safety*	148
3rd	Mansouri and Lurie (1993)	*American Journal of Hematology*	147
4th	Fan and Steinberg (1996)	*Regulatory Toxicology and Pharmacology*	138
5th	Barker et al. (1989)	*Anesthesiology*	137
6th	Ash-Bernal et al. (2004)	*Medicine*	108
7th	Curry (1982)	*Annals of Emergency Medicine*	95
8th	Guay (2009)	*Anesthesia and Analgesia*	84
9th	Fewtrell (2004)	*Environmental Health Perspectives*	83
10th	Nilsson et al. (1990)	*British Journal of Anaesthesia*	79

*SCR* standard competition ranking

**Table 5:** Top ten most highly productive institutions that published articles related to methaemoglobinaemia

SCR^a^	Institution, country	No. of documents (%)
1st	VA Medical Center, USA	16 (0.90)
2nd	The Institut National de la Santé et de la Recherche Médicale (INSERM), France	8 (0.45)
2nd	Universidade de Sao Paulo, Brazil	8 (0.45)
2nd	Belfast Health and Social Care Trust, UK	8 (0.45)
5th	Mayo Clinic, USA	7 (0.40)
5th	All India Institute of Medical Sciences, India	7 (0.40)
5th	Kyushu University, Japan	7 (0.40)
5th	University of Liverpool, UK	7 (0.40)
9th	Kanazawa University, Japan	7 (0.40)
9th	Duke University School of Medicine, USA	6 (0.34)
9th	Geisel School of Medicine at Dartmouth, USA	6 (0.34)
9th	University of Florida, USA	6 (0.34)
9th	Hopital Edouard Herriot, France	6 (0.34)
9th	Cleveland Clinic Foundation, USA	6 (0.34)
9th	Queen’s University Belfast, UK	6 (0.34)
9th	Fuzhou General Hospital PLA Nanjing District, China	6 (0.34)

*SCR* standard competition ranking

^a^Equal institutions have the same ranking number, and then a gap is left in the ranking numbers

## Discussion

In the current study, bibliometric indicators were used to evaluate the methaemoglobinaemia research output at the global level. The main strengths of this study are that it is the first bibliometric study on methaemoglobinaemia and it reveals the quantity and quality of methaemoglobinaemia-based research at the global level. In our study, publications related to methaemoglobinaemia first appeared in the 1940s and rapidly increased after 2000, with one-third of the articles (33.2 %) being published between 2000 and 2013. Scientific publications related to methaemoglobinaemia were similar to the general evolution in scientific research output observed over the last decade and especially in the last few years (Cheng and Zhang 2013; Delirrad et al. 2013; Lopez-Munoz et al. 2013; Sweileh et al. 2013; Zyoud et al. 2014a). Another aspect of interest for the current study in relation to the scientific publications related to methaemoglobinaemia is research paper quality. It should be noted that three of the top ten journals in which articles related to methaemoglobinaemia were published carry IFs greater than ten and have significant impacts in the field of medicine: *New England Journal of Medicine*, *Journal of the American Medical Association*, and *British Medical Journal*.

As shown in our study, the principal finding of this study is that in absolute terms of numbers of articles published in the field of methaemoglobinaemia, the USA is by far the largest contributor. This finding agrees with similar bibliometric studies (Zyoud et al. 2014b; Rymer and Choa 2015; Zyoud et al. 2015a, c). In countries such as the USA, UK, India, France, and Japan, the total output of scientific publications related to methaemoglobinaemia accounted for more than 40 % of global research output. This activity in the field of methaemoglobinaemia may be related to population size or socio-economic factors associated with these countries (Miro et al. 2009; Zyoud et al. 2015a). Furthermore, the number of active researchers, size of funding, prevalence of the disease in certain population, and research competition probably played a role in increasing the number of publications from these countries (Rahman and Fukui 2001, 2003; Man et al. 2004; Benamer and Bakoush 2009). The current study indicate that Israel and Turkey have produced the most research articles from the Middle East region, whereas India, Japan, and Australia are the major research contributors from the Asia–Pacific region, while the UK and France have produced the most research articles from Europe. The top ten most productive countries of this study comprise many countries that are familiar to other scientific productivity rankings (Essential Science Indicators 2012). We are unable to interpret these findings in light of other results, as we have not found any comparative studies. However, previous bibliometric studies have reported similar findings (Li et al. 2013; Ramos et al. 2013; Sweileh et al. 2014b).

The current study shows that the *h*-index of countries with a high number of internationally collaborated articles, such as the USA and the UK, is markedly higher than that of countries with a low number of internationally collaborated articles. Several studies showed that more international collaboration may lead to more influential publications due to shared research ideas and increased citations and visibility (Foley and Della Sala 2014; Freeman and Huang 2014; Li and Zhao 2015). The USA and UK achieved the highest *h*-index in the field of methaemoglobinaemia research, signifying a higher quality of research than other countries. The scientific research has shown an enormous interest in the *h*-index, as shown by the high number of publications on this subject (Wykes et al. 2013; Therattil et al. 2014; Zhang et al. 2015). The *h*-index allows differentiation of the scientific productivity of a researcher with objectivity and accordingly may play an important role when making decisions about awarding prizes, fund allocation, and promotion (Costas and Bordons 2007).

The articles that were published before 2000 were the most frequently cited. Articles that are highly cited are generally most read and referenced and likely to be influential publications within a particular field (Lipsman et al. 2014; Sharma and Lawrence 2014). Furthermore, that a publication is highly cited serves as an indication for its influence within a discipline (Cardona and Sanz 2014; Hsu and Ho 2014; Sharma and Lawrence 2014). The average number of citations per document obtained in our study is similar to that reported in toxicological journals but slightly less than that reported in other scientific journals (Bird 2008; Jang and Rusyniak 2011). Citations for publications in toxicology disciplines are usually low compared with those in other scientific disciplines (Zyoud et al. 2014a, c, 2015b, d).

This study has few limitations. Most were similar to recent bibliometric studies from our research group (Zyoud et al. 2014a, b, c, d, 2015a, c). First of all, some articles could not be found, mainly because the search was limited to keywords “methemoglobinemia” and “methaemoglobinaemia” in the title. Therefore, false negative results are a possibility. Additionally; the limitation of this study was in using the Scopus database alone; therefore it is possible that articles published in non-Scopus-cited journals may be missing from the analysis.

## Conclusions

This bibliometric analysis provides data contributing to a better understanding of the methaemoglobinaemia research field. The number of publications on methaemoglobinaemia increased significantly after 2000. The USA was the most productive country as measured by total publications. The USA and UK achieved the highest *h*-index in the field of methaemoglobinaemia research, signifying a higher quality of research than other countries. The present data reveal promising progress for research activity in the field of methaemoglobinaemia. Research activity and number of publications may be greatly enhanced by committing more to international collaborative research projects related to methaemoglobinaemia.

## Abbreviations

IFs: impact factors
JCR: Journal Citation Report
MetHb: methaemoglobin
SJR: SCImago Journal Rank
